# Clinical and GAA gene mutation analysis in mainland Chinese patients with late-onset Pompe disease: identifying c.2238G > C as the most common mutation

**DOI:** 10.1186/s12881-014-0141-2

**Published:** 2014-12-20

**Authors:** Xiao Liu, Zhaoxia Wang, Weina Jin, He Lv, Wei Zhang, Chengli Que, Yu Huang, Yun Yuan

**Affiliations:** Department of Neurology, Peking University First Hospital, Beijing, 100034 China; Respiratory Department of Internal Medicine, Peking University First Hospital, Beijing, 100034 China; Department of Medical Genetics, School of Basic Medical Sciences, Peking University Health Science Center, Beijing, 100191 China

## Abstract

**Background:**

Pompe disease is an autosomal recessive lysosomal glycogen storage disorder that has been reported in different ethnic populations which carry different common mutations of the acid alpha-glucosidase (*GAA*) gene. The *GAA* mutation pattern in mainland Chinese patients with late-onset Pompe disease is still not well understood.

**Methods:**

We presented the clinical and genetic characteristics of 27 mainland Chinese late-onset Pompe patients from 24 families.

**Results:**

*GAA* mutation analysis revealed 26 different mutations, including 10 that were novel. The allelic frequency of c.2238G > C (p.W746C) was found to be 27.08% in this patient group. Respiratory dysfunction was diagnosed in 10 of 11 patients who underwent pulmonary function evaluation, although only four required ventilator support at night.

**Conclusions:**

Our findings indicate that c.2238G > C (p.W746C) is the most common mutation in mainland Chinese late-onset Pompe patients, as observed in Taiwanese patients. The novel mutations identified in this study expand the genetic spectrum of late-onset Pompe disease, and the prevalence of respiratory dysfunction highlights the importance of monitoring pulmonary function in late-onset Pompe patients.

## Background

Pompe disease (glycogen storage disease type II, acid maltase deficiency, OMIM #232300) is an autosomal recessive lysosomal glycogen storage disorder caused by a deficiency of the lysosomal enzyme acid α-glucosidase (GAA). Pompe disease occurs in approximately 1 per 40,000 births [1], and patients are typically classified as early (infantile) or late-onset (childhood/juvenile/adult) according to the age of symptom onset. Patients with classical infantile-onset Pompe disease display a combination of generalized skeletal muscle weakness and cardiac hypertrophy that provoke cardiorespiratory failure and death within the first year of life [2]. Conversely, the late-onset form of Pompe disease exhibits a less severe phenotype with progressive proximal skeletal muscle weakness and respiratory muscle involvement. These nonspecific symptoms often make Pompe disease clinically difficult to differentiate from other neuromuscular diseases, but valuable evidence can be provided by measurement of decreased GAA activity, observations of vacuoles in muscle fibers on muscle biopsy, and genetic tests of the *GAA* gene [3,4].

This gene has been mapped to chromosome 17q25.2–q25.3; it contains 20 exons and the first amino acid is encoded by exon 2. Pathogenic sequence variations in *GAA* can lead to complete or partial loss of lysosomal GAA activity, and a close correlation exists between the functional GAA protein and clinical phenotype: the less residual GAA activity, the earlier the onset and greater the severity of the disease [5,6]. To date, over 400 different mutations have been described [see http://www.pompecenter.nl]. Some mutations appear with considerable frequency in distinct ethnic groups. For example, c.-32-13 T > G is the most common mutation in Caucasian patients with a frequency as high as 34–47% [4,7-11]. Conversely, c.1935C > A (p.D645E) and c.2238G > C (p.W746C) are common in Taiwanese patients [12]. Awareness of Pompe disease has been increasing in recent years, and more cases have been reported worldwide [13-15]. However, limited data have been published about mainland Chinese late-onset Pompe patients [16-18]. We herein present the clinical features and *GAA* mutation pattern of 27 patients from 24 unrelated families with late-onset Pompe disease from mainland China.

## Methods

### Subjects

Twenty-seven patients from 24 unrelated families (Table 1) who were diagnosed with Pompe disease at the Department of Neurology, Peking University First Hospital (Peking, China) from 2003 to 2013, were recruited in this study. None of the patients had consanguineous parents. Their confirmatory diagnosis was based on clinical features, biochemical assay, muscle pathology, and molecular tests. All patients gave their informed consent for this study, and ethical approval for the study was obtained from the health authority ethical committee of Peking University First Hospital.

**Table 1:** **Clinical, enzymatic, and molecular information of 27 Chinese patients w** **ith late-onset Pompe disease**

**No.**	**Gender/Age**	**Age of onset(years)**	**Age of diagnosis (years)**	**Initial symptoms**	**Family history**	**CK(IU/L)**	**Muscle pathology**	**lymphocyteGAA activity (pmol/punch/hr)** ^**1**^	**GAA gene mutation**		**Requirement of ventilator support**
									**Allele1**	**Allele2**	
1	F/3	1.2	3	Muscle weakness	No	568	Vacuolar myopathy	ND	c.503G > A	c.2237G > A	Yes
2	F/3.5	2	3.5	Muscle weakness and adynamia in swallowing	No	700	Vacuolar myopathy	ND	c.503G > C	c.1082C > T	No
3	M/14	8	14	Muscle weakness	No	907	Vacuolar myopathy	ND	c.796C > T	c.1309C > T	No
4	F/22	5	22	Muscle weakness	No	1101	Vacuolar myopathy	ND	c.1562A > T	c.1781G > A	No
5	M/30	10	30	Muscle weakness	No	642	Vacuolar myopathy	ND	c.503G > A	c.2237G > A	No
6	F/23	21	21	Limb girdle weakness	No	1047	ND	0	c.1355delC	c.2238G > C	Yes
7	M/17	17	17	Muscle weakness	No	1413	ND	ND	c.2238G > C	c.2238G > C	No
8	M/12	12	12	Muscle weakness	No	1365	Vacuolar myopathy	3.15	c.871C > T	c.2238G > C	No
9	M/23	21	21	Limb girdle weakness	No	2391.6	ND	2.25	c.323G > A	c.2014C > T	No
10	M/14	6	13	Respiratory insufficiency	No	637	Vacuolar myopathy	3.96	c.1935C > A	c.2238G > C	No
11	F/17	3	4	High level of serum CK	No	1227	Vacuolar myopathy	0.25	c.2238G > C	c.2662G > T	No
12	F/15	10	13	Limb girdle weakness	No	ND	Vacuolar Myopathy	4.04	c.2238G > C		No
13	F/24	12	20	Muscle weakness	No	1200	Vacuolar myopathy	1.15	c.1561G > A	c.2161G > T	Yes
14	F/25	15	23	Muscle weakness	No	662.6	Vacuolar myopathy	ND	c.1315_1317delATG	c.2238G > C	Yes
15	F/35	29	35	Muscle weakness	No	406	Vacuolar myopathy	3.54	c.1082C > T		No
16	F/33	32	32	High level of serum CK	No	927	Vacuolar myopathy	3.32	c.-32-13 T > G	c.2662G > T	No
17	M/32	28	32	Muscle weakness	No	117	Vacuolar myopathy	8.05	c.1634C > T	c.2662G > T	No
18	F/35	25	25	Sleep disordered breathing	No	79	Vacuolar myopathy	ND	c.2238G > C	c.2431delC	Yes
19	M/20	19	20	Limb girdle weakness	No	800	Vacuolar myopathy	3.46	c.1634C > T	c.1993G > A	Yes
20	F/23	23	23	Respiratory insufficiency	No	345	Vacuolar myopathy	5.98	c.1551 + 3_c.1551 + 6delAAGT	c.2238G > C	Yes
21	F/32	23	30	Muscle weakness	No	139	Vacuolar myopathy	1.1	c.1935C > A	c.2238G > C	Yes
22	F/14	9	14	Limb girdle weakness	No	970	Vacuolar myopathy	ND	c.2446G > A	c.2662G > T	No
23^2^	F/30	24	30	Muscle weakness	Yes	563	ND	ND	c.1396delG	c.2238G > C	No
24^2^	F/28	23	28	Muscle weakness	Yes	746	ND	ND	c.1396delG	c.2238G > C	No
25^3^	M/24	16	22	respiratory insufficiency and fatigue	Yes	790	ND	0	c.241C > T	c.2238G > C	Yes
26^3^	F/31	12	28	Muscle weakness	Yes	ND	ND	0.6	c.241C > T	c.2238G > C	No
27^3^	F/29	27	27	Limb girdle and muscle weakness	Yes	303	ND	0.44	c.241C > T	c.2238G > C	Yes

1 The median activities of 19 normal controls and 14 carriers were 36.37(range, 15.16–297.86) and 24.77 (range, 11.84–43.97) pmol/punch/hour, respectively.

2 patient 23 and 24 are siblings; 3 patient 25, 26, 27 are siblings.

Clinical data included disease history and physical examination. Eleven patients with the late-onset form of Pompe disease underwent a respiratory function evaluation, including measurement of forced vital capacity (FVC) in a sitting and supine position, forced expiratory volume in one second (FEV1), maximal inspiratory pressure (MIP), maximal expiratory pressure (MEP), and cough peak flow (CPF).

### Muscle pathology

Muscle biopsies were carried out in 19 patients. Muscle specimens were snap frozen in cooled isopentane, and then stored at −80°C until required for analysis. Cryostat sections were prepared and stained according to standard procedures with hematoxylin eosin, modified Gomori trichrome, periodic acid-Schiff (PAS), oil red O, adenosine triphosphatase, nicotinamide adenine dinucleotide-tetrazolium reductase (NADH-TR), succinate dehydrogenase, cytochrome c oxidase (COX), and non-specific esterase.

### Biochemical assays

In 15 of the 27 patients, GAA enzyme activity in dried blood spots (DBS) was determined with 4- methylumbelliferyl- alpha-D-glucoside (4-MUG) as the substrate and acarbose as an inhibitor of maltose glucoamylase (MGA) using a fluorometric assay as described [19].

### *GAA* mutation analysis

*GAA* mutation screening was performed in all patients. Genomic DNA was extracted from peripheral blood or frozen muscle biopsy specimens using standard procedures. All *GAA* exons and intron/exon boundaries were amplified by PCR, and then PCR products were purified and sequenced using an ABI 3730XL automatic sequencing machine (Applied Biosystems, Life Technologies, Carlsbad, CA, USA). Sequences were compared with the *GAA* reference DNA sequence (GenBank Accession: NM_000152) to identify pathogenic mutations. The cDNA was numbered with +1 corresponding to the A of the ATG translation initiation codon and with codon 1 as the initiation codon. The pathogenic nature of novel missense mutations was verified by direct sequencing of 100 unrelated healthy individuals. Additionally, *GAA* mutation analysis was performed in the parents of seven Pompe patients after obtaining their informed consent. To determine the effect of a possible splice-site mutation (c.1551 + 3_c.1551 + 6delAAGT) in intron 9, we extracted Total RNA from Patient 20′s muscle samples, and amplified the whole cDNA by reverse transcriptase-polymerase chain reaction (RT-PCR) using the SuperScript III First-Strand DNA Synthesis kit (Invitrogen, Carlsbad, CA, USA). A region encompassing exons 9–10 was amplified using primers G9-10 F: 5′- CGTTCAACAAGGATGGCTTC-3′ and G9-10R: 5′-GTGGGTTCTCCAGCTCATTG-3′. PCR products were analyzed by agarose electrophoresis.

## Results

### Clinical manifestations of patients

The clinical features of the 27 patients (9 male, 18 female) are summarized in Table 1. Patients 23 and 24 are a sibling pair, and patients 25, 26, and 27 are also siblings.

In present study, the patients’ age at onset ranged from 1.2–32 years with a median age of 21 years. Twenty-one (77.8%) complained of muscle weakness as their initial and main symptom. All patients had skeletal muscle weakness predominantly affecting the proximal extremities. Four patients reported that respiratory insufficiency was the initial clinical symptom that prompted them to seek medical help. Two patients presented with hyperCKemia as their initial symptoms. The median age at diagnosis was 22 years (range, 3–35 years). Eighteen patients had their diagnosis confirmed initially by muscle pathology, followed by mutation detection and/or GAA activity assay. Six patients were diagnosed by a combination of GAA activity assay and genetic analysis, and three patients by genetic analysis alone.

With disease progression, 15 late-onset patients suffered respiratory dysfunction, and persistent or intermittent assisted respiration was required in 10 of these (Table 1). The median serum creatine kinase (CK) level was 700 IU/L (range, 79.0–2,391.6 IU/L). The echocardiography performed in four late-onset patients showed no significant abnormality. Respiratory function evaluation in 11 late-onset patients (four requiring respiratory assistance at night) revealed that 90.90% (10/11) were abnormal. Further detailed clinical information has been reported by us previously [17].

### Muscle pathology

**Figure 1:**
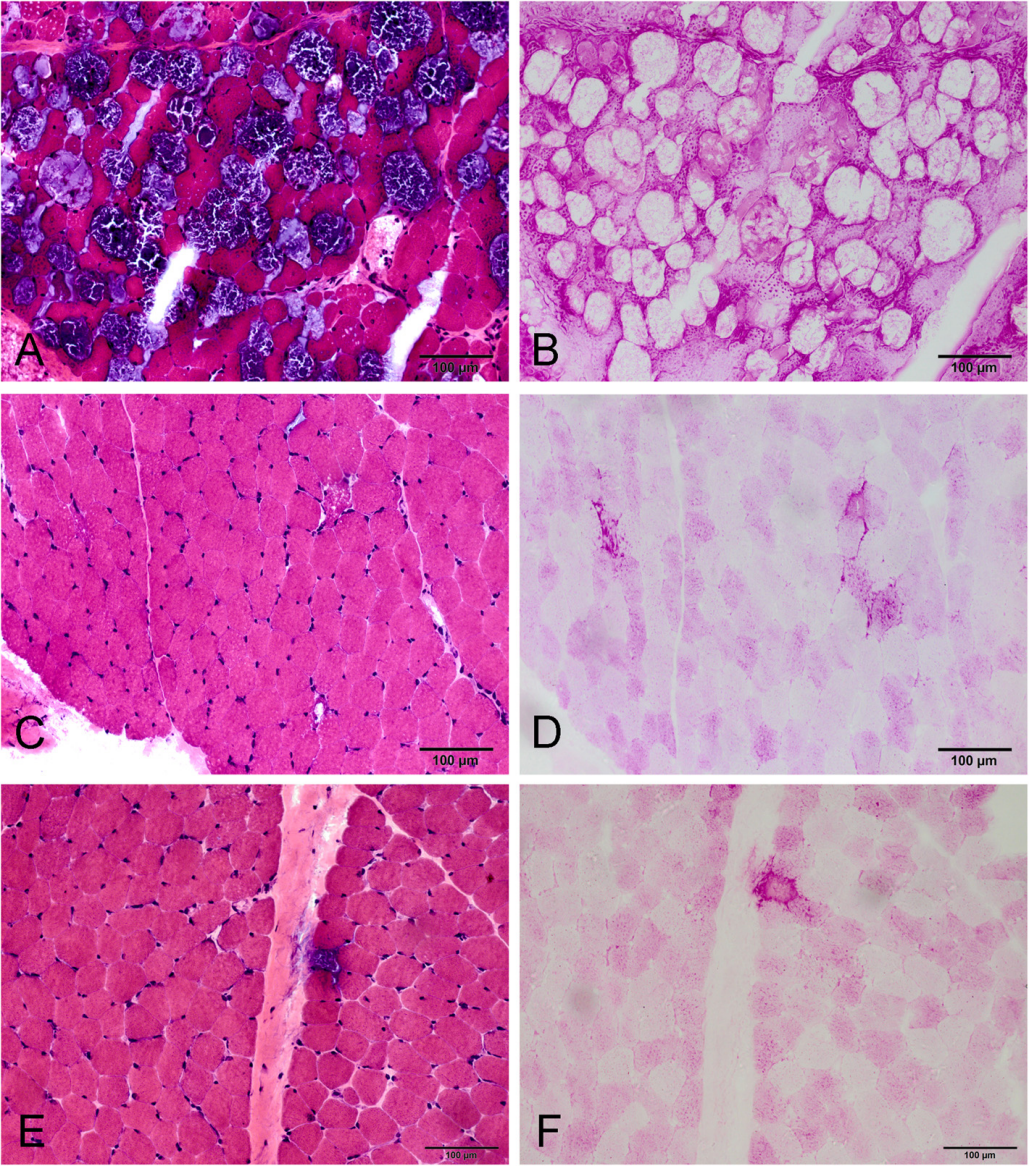
**Myopathological changes in Patient 2 (A and B), Patient 12 (C and D) and Patient 22 (E and F).** H&E staining shows extensive vacuolation in many fibers in Patient 2 **(A)**, but only a few vacuolar fibers in Patient 12 **(C)** and Patient 22 **(E)**. Vacuolar fibers stained positive for glycogen with PAS **(B, D and F)**.

All 19 patients who underwent muscle biopsy showed muscle fibers with vacuoles that stained positive for glycogen in a PAS stain. The proportion of vacuolar fibers was various among all the patients (Figure 1).

### GAA activity assay

The median GAA activity of 16 patients was 2.70 pmol/punch/hour (range, 0–8.05), while the median activities of 19 normal controls and 14 carriers were 36.37(range, 15.16–297.86) and 24.77 (range, 11.84–43.97) pmol/punch/hour, respectively.

### *GAA* mutations

**Figure 2:**
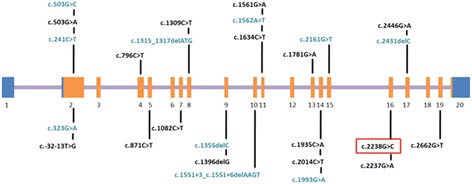
**GAA mutation spectrums in 27 Chinese late-onset Pompe patients.** All described mutations are shown above (blue, UTR; purple, introns; orange, exons).

Among the 27 patients recruited in this study there were five sibling pairs from two separate families, so 24 unrelated families are presented. *GAA* mutation analysis disclosed 21 families with compound heterozygous mutations, one with homozygous mutations, and two with only one heterozygous mutation (Table 1). A total of 26 different mutations were detected in the 24 families (Table 1, Figure 2), including 18 missense mutations, two nonsense mutations, four deletion mutations, and two splice site mutations. Fifteen patients from 12 families carried the c.2238G > C mutation, including 14 compound heterozygotes and one homozygote. The allele frequency of the c.2238G > C mutation in our patients was therefore 27.08% (13/48). Of the 26 mutations, 15 (c.503G > A, c.796C > T, c.871C > T, c.1082C > T, c.1309C > T, c.1561G > A, c.1634C > T, c.1781G > A, c.1935C > A, c.2014C > T, c.2238G > C, c.2662G > T, c.2237G > A, c.1396delG, and c.-32-13 T > G) have previously been reported as pathogenic, one (c.2446G > A) was reported as non-pathogenic[see http://www.pompecenter.nl], but the other 10 (c.323G > A, c.503G > C, c.1562A > T, c.1993G > A, c.241C > T, c.2161G > T, c.1355delC, c.1315_1317delATG, c.2431delC, and c.1551 + 3_c.1551 + 6delAAGT) are novel.

**Figure 3:**
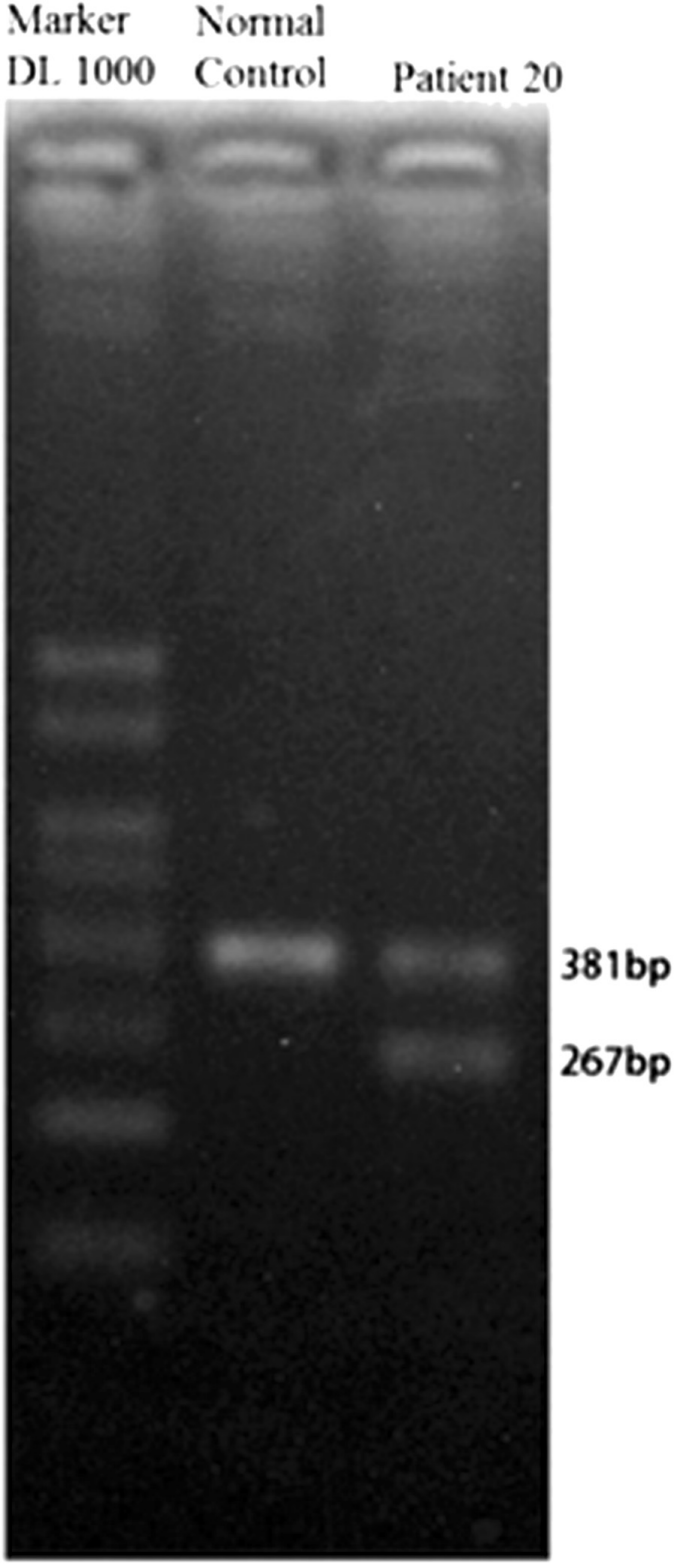
**Exon 10 skipping in Patient 20.** Muscle cDNA was amplified with the primers encompassing exon 9 and 10 that normally yield a 381-bp fragment. An additional 267-bp fragment was detected in Patient 20. The 114-bp difference is exactly the same with the size of exon 10.

**Figure 4:**
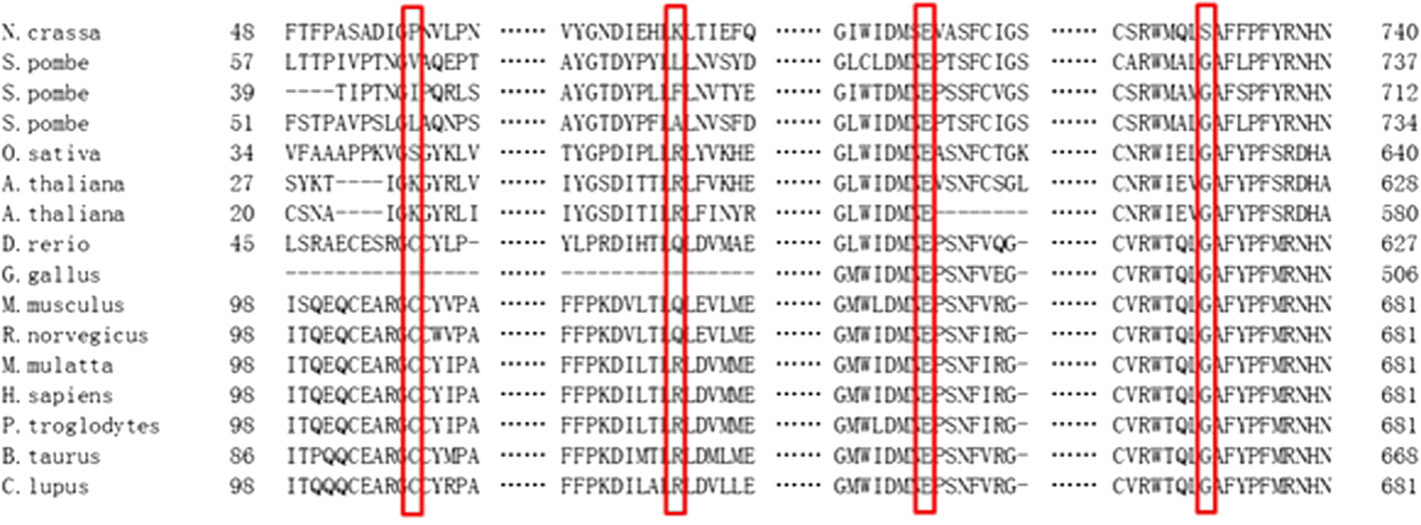
**Conservation of four novel missense mutations in different species.**

None of the 10 novel mutations were detected in 100 unrelated healthy controls. The c.241C > T and c.2161G > T mutations lead to a premature stop in protein synthesis, which was assumed to be deleterious since stop codons located upstream of the main stop codon could result in truncated protein. There were four novel small deletions detected, among them c.1551 + 3_c.1551 + 6delAAGT confirmed to cause exon 10 skipping by RT-PCR (Figure 3), c.1355delC, and c.2431delC mutations predicted to cause a frame shift effect, while c.1315_1317delATG predicted to cause in frame deletion. Of the four novel missense mutations, the amino acids mutated in p.Glu521Val (c.1562A > T) and p.Gly665Arg (c.1993G > A) are highly conserved across the examined species (Figure 4), suggesting that they are potentially pathogenic. p.Cys108Tyr (c.323G > A) and p.Arg168Pro (c.503G > C), however, were less conserved, indicating that the mutations are more likely to be mild in severity. The healthy parents of patients 11 (c.323G > A; c.2014C > T) and 21 (c.1634C > T; c.1993G > A) were found to carry one heterozygous mutation each that were present in their offspring.

## Discussion

Although Pompe disease is rare, it has been reported in a number of different ethnic populations, such as Caucasian, Taiwanese, Korean and Japanese [4,7-12,20-23]. This study is the largest series of mainland Chinese late-onset Pompe patients, including 27 patients from 24 unrelated families. Our results showed that the majority of our patients (15/27, 55.56%) carried the c.2238G > C mutation of *GAA*, and that the allele frequency of c.2238G > C was as high as 27.08%, making it the most common mutation in this group. This result is consistent with findings in Taiwanese Pompe patients [12], but different from the common mutation (c.1935C > A) in mainland Chinese infantile-onset group [24].Meanwhile, c.-32-13 T > G, the most common mutation of Caucasian origin with a frequency of 34–79% [3,4,7-10], was found in only one patient with compound heterozygous mutations in the present study. Other common mutations reported in certain populations, such as c.1316 T > A and c.1857C > G with a frequency of 36.6% in Korean patients [20], c.1064 T > C which is the predominant mutation in Portuguese patients [21], c.1726G > A with a frequency of up to 27.59% in Japanese patients [22], and the African-American mutation c.2560 C > T [23], were not detected in our patients. Our study therefore further supports the findings that different populations have different mutation hotspots.

As c.1935C > A, the other common mutation in Taiwan, was detected in only one patient from our group, and additionally, c.1726 G > A and c.2065G > A pseudodeficiency mutations which are common in Taiwanese, were absent in the present study, this suggests that the spectrum of *GAA* mutation differs not only between ethnicities but also from region to region in the same population. The absence of c.[2238G > C; 1726G > A] haplotype in our patients raises the possibility that the c.2238G > C mutation might have a different ancestor in Taiwan and mainland China. Further research involving more patients is needed to confirm this.

The diagnosis of this patients group with late-onset Pompe disease depended on the combination of clinical manifestations, muscle biopsy, blood-based GAA activity assay and GAA gene analysis. The mean age of onset the patients in the current study was 17.41 ± 8.99 years, which is younger than a previous investigation of a Caucasian background, and is in keeping with the study in Taiwanese Pompe patients [3,10]. The difference in onset age between Chinese and Caucasian patients with Pompe disease may be caused by the prevalence of the c.2238 G > C mutation and the low frequency of c.-32-13 T > G in the present set of patients, as different mutations have different effects on enzyme activity [12]. Muscular weakness was the most common initial symptom in our study, as seen in an earlier work; [25] however, it is noteworthy that respiratory impairment was very common in our late-onset patients, which is in contrast to the study in Germany in which no patients had respiratory symptoms [25].

Dyspnea without limb weakness was the first reported symptom in four of the current patients, while 10/27 (37.0%) of late-onset patients needed mechanical ventilation within 2.5 years of disease onset. Moreover, in the seven patients without mechanical ventilation, pulmonary function evaluation revealed decreased pulmonary function in six. Together, our data support previous findings that monitoring pulmonary function is essential in late-onset Pompe disease to evaluate the need for mechanical ventilation [1,26,27].

Notably, in 18 of 27 late-onset patients, diagnosis of Pompe disease was confirmed initially by muscle pathology. Although a blood-based assay has been widely recommended as a simple diagnostic method, we still consider muscle biopsy to be a very useful tool as the diagnosis of Pompe disease can be challenging because of its heterogeneous clinical presentation and considerable overlap of signs and symptoms found in other neuromuscular diseases [28,29]. This is particular important in east Asia, where the high frequency of the p.[G576S;E689K] pseudodeficiency mutation in these ethnic populations can give false positive results of GAA activity. Moreover, it is not uncommon that only one pathogenic heterozygous mutation is detected in coding region of GAA gene in Pompe patients, as in patient 12, 15, and 22 of this group. In such cases, muscle pathology can provide solid evidence for disease diagnosis (Figure 1, Table 1). However, it should mentioned that the muscle biopsy still has its limitation in diagnosing Pompe disease due to the heterogeneity of muscle involvement, especially in patients with late onset Pompe disease [30].

## Conclusions

Our findings indicate that c.2238G > C (p.W746C) is the most common mutation in mainland Chinese late-onset Pompe patients, as observed in Taiwanese patients. The novel mutations identified in this study expand the genetic spectrum of late-onset Pompe disease, and the prevalence of respiratory dysfunction highlights the importance of monitoring pulmonary function in late-onset Pompe patients.
